# Modified Epiblepharon Repair Preserving Orbicularis Oculi Muscle

**DOI:** 10.1155/2022/8469812

**Published:** 2022-07-11

**Authors:** Hyun Chul Youn, Seunghwan Lee, Ju-Hyang Lee

**Affiliations:** ^1^Department of Ophthalmology, Ulsan University Hospital, University of Ulsan College of Medicine, Ulsan, Republic of Korea; ^2^Department of Ophthalmology, Yeongdeok Asan Hospital, University of Ulsan College of Medicine, Yeongdeok, Seoul, Republic of Korea; ^3^Department of Ophthalmology, Ajou University Hospital, Ajou University of Medicine, Suwon, Republic of Korea

## Abstract

**Methods:**

A retrospective review of was conducted of patients who received modified epiblepharon repair preserving orbicularis oculi muscle from April 2016 to October 2018. Removal of the orbicularis oculi muscle was minimally performed with eyelash rotating sutures and epicanthal weakening procedure. The preoperative severity of epiblepharon was classified according to skin fold height and cilia-corneal touch. Main postoperative outcomes were evaluated by functional success and cosmetic satisfaction.

**Results:**

A total of 208 eyelids of 104 patients were evaluated. The mean age was 7.2 ± 4.2 years with a mean follow-up time of 5.6 ± 4.6 months. Functional success was obtained in 206 eyelids (99.0%). Only two cases that had a total epiblepharon score of 7 showed a recurrence of mild cilia-corneal touch, but reoperation was not required. The cosmetic satisfaction score was 8.7 ± 1.8 (range, 1–10). The excellent cosmesis group with a cosmetic score of 9 or higher had significantly higher skin fold height (*p*=0.027).

**Conclusions:**

Modified epiblepharon repair preserving orbicularis oculi muscle can be effective in the treatment of lower epiblepharon regardless of its severity. Excellent outcomes were obtained functionally and cosmetically without debulking of the orbicularis oculi muscle.

## 1. Introduction

Epiblepharon is a common eyelid anomaly especially in Asian children [1]. It is characterized by a horizontal fold of redundant skin and the underlying orbicularis muscle that overrides the eyelid margin and pushes the cilia toward the cornea, resulting in ocular irritation and keratitis [2, 3]. Epiblepharon usually involves the medial half of the lower eyelid [1]. The pathogenesis of epiblepharon includes failure of the attachment of the eyelid retractor fibers to the anterior lamella [4], hypertrophy of the orbicularis oculi muscle [5], and medial epicanthal fold [6].

Several surgical procedures have been proposed for the repair of epiblepharon, including the modified Hotz procedure and cilia rotational suture with minimal skin excision [7].

Conventional repairs of the epiblepharon have focused on debulking of the excessive skin and orbicularis muscle [5, 7]. However, these procedures result in volume reduction and can lead to flat eyelids and a sunken appearance. Although these procedures can correct cilia-corneal touch and keratopathy in most patients, cosmetic configuration is an additional concern due to ethnic differences. Currently, pretarsal fullness is considered appealing and promoting youthfulness in many Asian people [8, 9]. Despite the postoperative functional success, patients and parents may have cosmetic dissatisfaction. For lower blepharoplasty, techniques have recently moved from volume reduction toward volume preservation or restoration [9–11]. In Asia, augmenting the pretarsal area is a growing trend. Pretarsal augmentation using hyaluronic acid, acellular dermal matrix, or fascia lata, has recently become popular with young Asian women [12, 13]. Although the significance of the augmented lower eyelid surgery has been emphasized in women, volume preservation is also important cosmetically for children and their parents.

To our knowledge, there has been no report emphasizing pretarsal fullness in the treatment of lower epiblepharon. Here, we report the cosmetic and functional outcomes of lower epiblepharon repair with preservation of the orbicularis oculi muscle.

## 2. Materials and Methods

We reviewed the medical records of all patients who underwent modified epiblepharon repair between April 2016 and October 2018. All procedures were performed by a single surgeon (J.H.L.) at the Ulsan University Hospital, Ulsan, Korea. This study was approved by the Institutional Review Board of the Ethics Committee under the tenets of the Helsinki Declaration.

Surgical indications were significant lower lid epiblepharon with obvious cilia-corneal touch which was causing irritative symptoms. The analysis excluded patients who were followed for less than 3 months after surgery and underwent concurrent medial epicanthoplasty. Photographs were obtained at each follow-up visit at 1 week, 1 month, 3 months and, if necessary, 6 months intervals thereafter.

**Figure 1:**
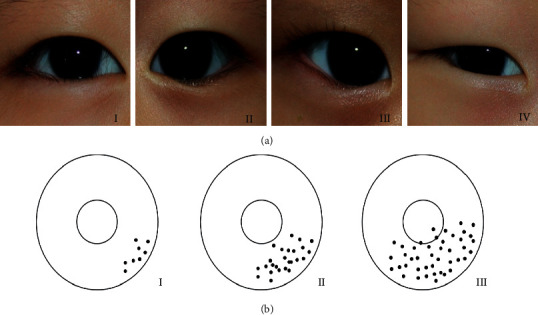
Preoperative classification of the lower lid epiblepharon according to the skin fold height: (a) The highest line of the horizontal skin fold below the lower lid margin was graded as class I, on the lower lid margin without concealment of the lid margin was graded as class II, above the lower lid margin with concealment of less than the medial one-third of the lid margin was graded as class III, and above the lower lid margin and concealing more than the medial one-third of the lid margin was graded as class IV. The grading of the cilia-corneal touch: (b) The cilia-corneal touch was class I if it was touching less than the medial one-third of the cornea; class II, less than the medial two-thirds of the cornea; class III, more than two-thirds of the cornea.

All patients were classified according to the severity of epiblepharon based on Khwarg's classification [14] by two examiners (SL and JL). The skin fold height and the area of the cilia-corneal touch were examined with a slit lamp ophthalmoscope. The height of the skin fold was classified into four groups by relation to the lower lid margin (Figure 1(a)). The severity of cilia-corneal touch was grouped into three classes according to the range of contact with the cornea in the primary position (Figure 1(b)). Total epiblepharon grades were calculated by adding the grades of the height of the skin fold and cilia-corneal touch, ranging from 2 to 7.

Functional success was defined as a correction of cilia-corneal touch and improvement of subjective symptoms. Cosmetic outcomes were evaluated by the preservation of pretarsal fullness with primary position and in smiling by reviewing photographs. Parents or patients received questionnaires about the cosmetic outcome in terms of pretarsal fullness after surgery. A numeric rating scale scored subjective cosmesis from 1 to 10. Unsatisfactory cosmesis was defined as 6 or less, and satisfactory cosmesis was defined as 7 or more. Investigators rated an “excellent” cosmesis score as 9 or 10, and the rest were classified as “nonexcellent.” We compared the clinical characteristics in two ways: the satisfactory group and the unsatisfactory group in terms of cosmesis, or the excellent and nonexcellent cosmesis.

Statistical analysis was performed using the SPSS software version 18.0 (SPSS, Chicago, Illinois, USA). Independent *t*-test and chi-square test for trend were used to evaluate the unsatisfactory cosmesis and preoperative severity of epiblepharon or body mass index (BMI). A *p* value <0.05 was considered statistically significant.

### 2.1. Surgical Procedure

**Figure 2:**
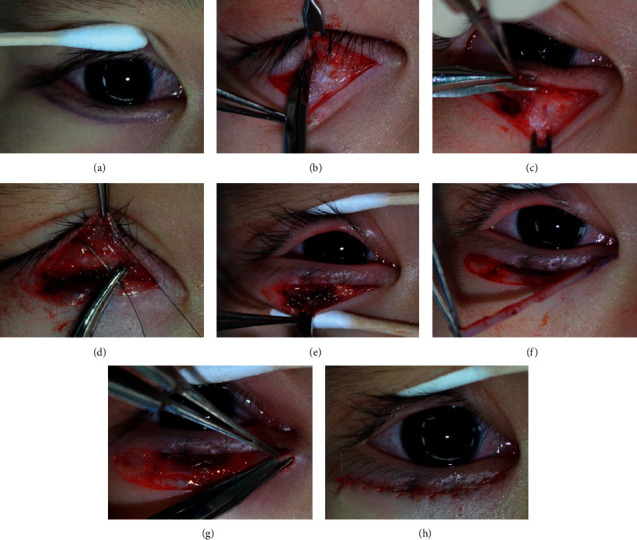
Modified epiblepharon repair preserving orbicularis oculi muscle. Skin incision line was made 1-2 mm below the ciliary line just below the lower punctum with lateral extension (a) Dissection was performed between the tarsus and the pretarsal orbicularis muscle (b) A small amount of pretarsal orbicularis muscle was excised (c) Eyelash rotating sutures were placed between the subcutaneous tissue of the upper skin flap and the tarsal plate in three to five points with 7–0 nylon sutures (d) To preserve the pretarsal orbicularis muscle, the skin flap was undermined carefully (e) An excess skin flap was excised (f) Medial dense fibrotic tissue and orbicularis muscle were removed under the skin flap (g) The skin was closed in a continuous manner (h).

All procedures were performed under general anesthesia. The incision was made 1-2 mm below the ciliary line horizontally from just below the lower punctum with lateral extension (Figure 2(a)). The lower eyelid was injected transcutaneously with a mixture of 2% lidocaine and epinephrine 1 : 100,000 for hemostasis. The skin was incised with a No. 15 Bard-Parker blade. Dissection was performed between the tarsus and the pretarsal orbicularis muscle with Westcott scissors (Figure 2(b)), and a small amount of pretarsal orbicularis muscle was excised to expose the tarsus and to make firm fixations with subcutaneous tissue of the upper skin flap (Figure 2(c)). Three to five eyelash rotating sutures were placed between the subcutaneous tissue of the upper skin flap and the exposed tarsus with 7–0 nylon sutures in a buried manner (Figure 2(d)). The excess skin-muscle flap was draped over the lower lid margin and marked with a surgical pen. A medial skin incision was extended to the medial canthus with a blunt angle. The skin flap was vertically reflected carefully beyond the intended line for skin excision to preserve pretarsal orbicularis muscle (Figure 2(e)). After skin flap elevation, excess skin was excised (Figure 2(f)). In case of orbicularis muscle hypertrophy, some lateral orbicularis oculi muscle was trimmed to prevent dog-ear. The nasal skin was pulled medially, and the dense fibrotic tissue and orbicularis muscle were carefully removed under the skin flap for weakening medial epicanthal fold (Figure 2(g)). Lateral dog-ear deformity was trimmed with a triangle shape. The preserved preseptal orbicular muscle was overlapped, and the skin was closed in continuous manner using a 6-0 fast absorbable plain gut suture (Figure 2(h)). Antibiotic ointment was applied over the skin incision for 1-2 weeks postoperatively.

## 3. Results

A total of 208 eyelids of 104 patients were included in this study; 46 patients (44.2%) were male, and 58 patients (55.8%) were female. All patients were Asian and underwent bilateral procedures. The mean age was 7.2 ± 4.2 (range, 3–26) years and mean follow-up periods were 5.6 ± 4.6 (range, 3–30) months (Table 1). Preoperative classification of epiblepharon was as follows by skin fold height: class I (*n* = 25, 12.0%), class II (*n* = 64, 30.8%), class III (*n* = 54, 26.0%), and class IV (*n* = 65, 31.3%). The preoperative cilia-corneal touch was classified as class I (*n* = 32, 15.4%), class II (*n* = 73, 35.1%), and class III (*n* = 103, 49.5%) respectively. The total epiblepharon score was 5.1 ± 1.4 (range, 2–7) (Table 2).

Good anatomical success was achieved in 206 eyelids (99.0%) following modified epiblepharon repair preserving the orbicularis muscle. Two eyelids, one in each of two individuals (1.0%), which showed a total epiblepharon score of 7 had a recurrence after surgery. The preoperative total scores of epiblephron were 5.1 ± 1.4 in the success group and 7.0 in recurred cases (*p*=0.047). All recurred cases were mild and cilia-conjunctival touches occurred only at the nasal side of the lower eyelids. Therefore, no reoperations were required.

Cosmetic outcome score was 8.7 ± 1.8 (range, 1–10) in 83 patients who consented to and completed the questionnaire at last follow-up. Excellent cosmesis score was achieved in 53 patients (64.6%). Unsatisfactory cosmetic outcome scores of 6 or less were found in seven patients (8.5%). When the satisfactory and unsatisfactory groups were compared based on the cosmesis score of 7, there was no significant difference in age, BMI, and severity of the epiblepharon. Although not statistically significant, the skin fold height (2.7 ± 1.0 vs. 3.1 ± 0.9, *p*=0.262) and severity of epiblepharon (4.9 ± 1.4 vs. 5.4 ± 1.3, *p*=0.359) tended to be high in the unsatisfactory group. However, in the comparison of the excellent and nonexcellent groups based on scores of 9, skin fold height was significantly higher in the nonexcellent group (2.6 ± 1.0 vs. 3.1 ± 0.9, *p*=0.027) (Tables 3 and 4).

The mean preoperative BMI was 18.1 ± 3.3 (range, 13.6–29.8) kg/m^2^, which was 17.9 ± 3.0 kg/m^2^ in the satisfactory group and 18.8 ± 4.2 kg/m^2^ in the unsatisfactory group in terms of cosmesis (*p*=0.584).

Postoperative complications included suture granuloma (6 eyelids, 2.9%), inclusion cyst (4 eyelids, 1.9%), suture abscess (2 eyelids, 1.0%), and suture exposure (1 eyelid, 0.5%). All complications were controlled with antibiotic ointment and simple removal of exposed sutures or inclusion cysts.

## 4. Discussion

In our study, anatomical success without recurrence was achieved in 206 eyelids (99.0%). A total of 91.5% of patients and parents were satisfied in terms of pretarsal fullness and cosmetic outcomes at the last follow-up.

Various surgical procedures have been utilized for epiblepharon repair. Incisional techniques include lid crease and capsulopalpebral fascia repair [15], modified Hotz procedure [16, 17], cilia rotational suture with minimal skin excision [7], and combined surgery with epicanthoplasty [18–20]. Full-thickness everting suture technique [21] was introduced as a non-incisional surgery, but despite its simplicity, the recurrence rate was high [21, 22].

Surgical techniques have previously focused on the excision of excessive skin and muscle. The anterior lamellar redundancy is not the main contributing factor, but rather a possible aggravating factor to epiblepharon [2]. Hypertrophy of the orbicularis oculi muscle has not been verified by microscopic studies [23]. The concept was established that debulking of the excessive skin and orbicularis muscle during the operation was not crucial for the repair of epiblepharon. Although these surgeries have resulted in favorable anatomical success, lower eyelid crease or ectropion can be encountered, which is not a cosmetic feature favored by Asians [1, 7]. Therefore, the surgical paradigm shifted to eyelash everting sutures from Hotz operation [1]. In recent decades, the eyelash rotational suture technique has been widely used in the correction of epiblepharon [7, 19, 20, 24]. Despite successful repair of epiblepharon, patients or their guardians may not be satisfied with the cosmetic results such as a sunken or hollowed appearance or flatness of the eyelid.

Pretarsal fullness of the lower eyelid is caused by layers of rolled muscle becoming narrower and thicker when smiling. Overriding on the pretarsal part of the preseptal orbicularis muscle is prominent in the Asian lower eyelid and causes the development of eyelid fullness [25]. Interestingly, it may result in indistinct lower eyelid crease and epiblepharon [26]. For this reason, excision of the redundant orbicularis oculi muscle has been considered an essential surgical step.

The lower eyelid in Asian individuals is different from Caucasians in terms of eyelid fullness and no eyelid crease [27]. The microscopic anatomy shows overriding on the pretarsal part of the preseptal orbicularis muscle in Asian lower eyelids [25]. There are likely unique differences in eyelid anatomy within each ethnicity. For most Asians, eyelid fullness is considered attractive and desirable [10, 13, 28]. However, eyelid fullness might be an unacceptable and less attractive result by a Caucasian individual's standards of aesthetics, which may be a cultural difference [28]. Understanding these ethnical differences is a crucial step toward obtaining satisfactory surgical outcomes.

In the field of cosmetic eyelid procedures, the paradigm has recently shifted to volume preservation or augmentation. Most procedures or surgeries for augmentation of the pretarsal area have been performed for cosmetic purposes in Asian countries [9, 10, 13]. In terms of these cultural differences, we initiated this study because it is important to maintain eyelid fullness after epiblepharon repair for aesthetic reasons.

Several studies have shown that a higher BMI may be one of the aggravating factors of epiblepharon [29, 30]. In our study, 14 obese patients with a BMI of 25 or higher all achieved functional success. Even in the recurred 2 cases with medial trichiasis, BMIs were 18.8 and 15.8 kg/m^2^. Our study showed that BMI tended to be higher in the cosmetically unsatisfactory group with a cosmetic score of 6 or less; however, this was not statistically significant. In our study, for patients with a higher skin fold height, functional success and general cosmesis were achieved, yet excellent cosmesis was not obtained. When determining the amount of resection, a small amount of orbicularis resection is recommended if the skin fold height is high rather than based on BMI or obesity.

Notably, we also achieved a high anatomical success rate (94.7%, 36/38) even in severe epiblepharon patients with a class IV skin fold height and class III corneal touch. Our study revealed that even with severe epiblepharon, modified epiblepharon repair with preservation of the orbicularis oculi muscle achieved excellent success both functionally and cosmetically.

Based on this study, there are two critical steps that can improve surgical success even with muscle preservation: meticulous eyelash rotating sutures and medial epicanthal weakening procedure. In previous reports, the most common cause of surgical failure after conventional techniques was relapse of the medial cilia touch. Epicanthal fold is an obvious characteristic of Asian individuals, which may serve to deteriorate the epiblepharon in the medial half of the eyelid [6, 17]. Many surgeons have introduced various surgical methods accompanied by medical epicanthoplasty to reduce recurrence [1, 18, 19]. However, medial epicanthoplasty is time-consuming and inevitably accompanied by scarring. Asamura et al. [17] also reported that medial epicanthoplasty was not necessary for the correction of the epiblepharon. However, we performed simple medial epicanthal weakening using Sa's technique [20] to improve surgical success.

The limitations of this study are that it is a retrospective design and single-institution study. Moreover, the cosmetic outcome was conducted only with the patient/guardian's satisfaction survey. However, the study is valuable in that to our knowledge this is the first time that a study has considered cosmesis of the pretarsal fullness as well as anatomical success in epiblepharon surgery.

In conclusion, the modified epiblepharon repair which involves preserving the orbicularis oculi muscle is an effective procedure for the treatment of lower epiblepharon regardless of its severity. Without debulking the orbicularis oculi muscle, we achieved excellent functional outcomes with cosmetic satisfaction accompanied by pretarsal fullness. To achieve highly excellent cosmesis, a small amount of anterior lamellar resection might be helpful in patients with a high skin fold height.

## Figures and Tables

**Table 1:** Characteristics of the 104 participating patients (208 eyes).

Mean age (years)	7.2 ± 4.2 (3–26)

Sex	
Male	46 (44.2%)
Female	58 (55.8%)

BMI^*∗*^ (kg/m^2^)	18.1 ± 3.3 (13.6–29.8)

Mean duration of the follow-up period (months)	5.6 ± 4.6 (3∼30)

^*∗*^BMI, body mass index.

**Table 2:** Preoperative classification of epiblepharon.

Cilia-corneal touch, *n* (%)	Skin fold height, *n* (%)				Total
	I	II	III	IV	
I	8	11	9	4	32 (15.4)
II	5	26	19	23	73 (35.1)
III	12	27	26	38	103 (49.5)
	25 (12.0)	64 (30.8)	54 (26.0)	65 (31.3)	208

**Table 3:** Correlation of postoperative cosmetic satisfaction with clinical characteristics and the preoperative severity of epiblepharon.

Cosmesis score	Cosmetically satisfactory (*n* = 75)	Cosmetically unsatisfactory (*n* = 7)	*p* value	Excellent cosmesis (≥9) (*n* = 53)	Nonexcellent cosmesis (*n* = 29)	*p* value
	≥7	<6		≥9	≤8	
Age	6.9 ± 3.8	8.3 ± 2.9	0.268	7.2 ± 4.3	6.6 ± 2.6	0.455

BMI	17.9 ± 3.0	18.8 ± 4.2	0.584	17.5 ± 2.7	18.8 ± 3.7	0.124

f/u	5.2 ± 4.3	7.0 ± 2.6	0.122	5.4 ± 5.0	5.1 ± 2.4	0.638

Grade of epiblepharon						
Skin fold height	2.7 ± 1.0	3.1 ± 0.9	0.262	2.6 ± 1.0	3.1 ± 0.9	0.027^*∗*^
Corneal touch	2.2 ± 0.8	2.3 ± 0.8	0.849	2.3 ± 0.7	2.1 ± 0.8	0.266
Severity score	4.9 ± 1.4	5.4 ± 1.3	0.359	4.9 ± 1.3	5.2 ± 1.4	0.340

By independent *t*-test. ^*∗*^Significantly associated parameter.

**Table 4:** Cosmetic satisfactions depending on the skin fold height and cilia-corneal touch.

	Skin fold height				*p* value	Corneal touch			*p* value
	I	II	III	IV		I	II	III	
Cosmetically satisfactory (n = 75, (%))	10 (13.3)	23 (30.7)	21 (28.0)	21 (28.0)	0.277	15 (20.0)	28 (37.3)	32 (42.7)	0.844
Cosmetically unsatisfactory (n = 7, (%))	0	2 (28.6)	2 (28.6)	3 (42.9)		1 (14.3)	3 (42.9)	3 (42.9)	
Excellent cosmesis (n = 53, (%))	9 (17.0)	17 (32.1)	15 (28.3)	12 (22.6)	0.032^*∗*^	9 (17.0)	19 (35.8)	25 (47.2)	0.509
Nonexcellent cosmesis (n = 29, (%))	1 (3.4)	8 (27.6)	8 (34.8)	12 (41.4)		7 (24.1)	12 (41.4)	10 (34.5)	

By chi-square test for trend. ^*∗*^Significantly associated parameter.

## Data Availability

The data are restricted access due to patient privacy. It can be accessed by requesting through the Institutional Review Board of Ulsan University Hospital (IRB No. 2017-11-016).

## Additional Points

Modified epiblepharon repair preserving orbicularis oculi muscle is an effective treatment for epiblepharon regardless of its severity. Without debulking of the orbicularis oculi muscle, excellent cosmetic and functional outcomes were obtained with preservation of the pretarsal fullness.
